# Factors affecting advance directives completion among older adults in Korea

**DOI:** 10.3389/fpubh.2024.1329916

**Published:** 2024-02-02

**Authors:** Seunghye Choi, Hana Ko

**Affiliations:** College of Nursing, Gachon University, Yeonsu-gu, Incheon, Republic of Korea

**Keywords:** advanced directives, dying-well, advanced care planning, aged, end of life

## Abstract

**Objective:**

Advance directives (ADs) provide an opportunity for patients to enhance the quality of their end-of-life care and prepare for a dignified death by deciding treatment plans. The purpose of this study was to explore the multiple factors that influence the advance directives completion among older adults in South Korea.

**Methods:**

This was a secondary analysis of a cross-sectional study of 9,920 older adults. The differences in ADs based on subjects’ sociodemographic characteristics, health-related characteristics, and attitude toward death were tested using the chi-squared and *t*-test. A multinomial logistic regression model was used to identify the influencing factor of ADs.

**Results:**

The number of chronic diseases, number of prescribed medications, depression, insomnia, suicide intention, and hearing, vision, or chewing discomfort were higher in the ADs group compared to the non-ADs group. The influencing factors of the signing of ADs included men sex, higher education level, exercise, death preparation education, lower awareness of dying-well, and experience of fracture.

**Conclusion:**

Information dissemination regarding ADs should be promoted and relevant authorities should consider multiple options to improve the physical and psychological health of older adults, as well as their attitude toward death to increase the ADs completion rate.

## 1 Introduction

Since 2017, Korea has become an aged society, with the proportion of the population aged 65 or above exceeding 14% (1). With the rapid aging of the population, the support for older adults, which was the traditional function of the family, has weakened. Studies have revealed that many individuals die after receiving life-sustaining treatment and care for an extended period in various care settings, such as nursing facilities, nursing hospitals, and acute care hospitals (2, 3). According to a 2022 report, 14.9% of older adults aged 65 or above died at home, and 76.4% died in medical institutions including nursing hospitals (4).

The concept of dying-well refers to a dynamic and continuous process in which patients, their families, and medical staff interact to fulfill patients’ wishes during the end-of-life preparations (5). The family interactions include avoidance of futile prolongation of life, death with dignity and comfort, and experiencing positive feelings with the family (5). In a previous study conducted in the Republic of Croatia, the presence of family and loved ones was also a significant affecting factor in the good death (6).

Advance directives (ADs) are a significant component of life-care planning. They are legal documents through which a person aged 19 or above with decision-making ability makes a written decision in advance about the medical treatment to be received in the future according to his/her own values after being provided sufficient information through an advance directive on life-sustaining treatment (7). ADs were developed by an American lawyer, Luis Kutner to make a legal statement about one’s healthcare, much like about one’s wealth and property (8). ADs have dramatically evolved over time, comprising the living will, Durable Power of Attorney, Do Not Resuscitate forms, and Medical Orders for Sustaining Treatment (9, 10).

ADs were introduced as an opportunity for patients to enhance the quality of their end of-life care and prepare for a dignified death by discussing and deciding treatment plans with their families and medical staff (11). Owing to these advantages, one in three US adults have documented their ADs (12). In February 2018, the Act on Decisions on Hospice Palliative Care and Life-Sustaining Treatment for Patients in the Ending Process was enforced in Korea. However, seven out of 10 cases of withholding or discontinuing life-sustaining treatment in Korea are family decisions (13). Even in the West, where ADs were early legislated, it has been reported that the completion of ADs remains a social, cultural, and ethical dilemma so it should be utilized effectively (14). In Korea, which is a family-oriented culture, the factors that influence the completion of ADs, which is a legislated form, may be different from those in the West (15).

In Korea, ADs introduction and research are in the early stages. Existing studies have mainly explored the knowledge, attitude, and intention toward the medical staff or ADs of adults. Moreover, the entities responsible for registering advance directives for life-sustaining treatment in Korea include medical institutions, non-profit organizations, and public institutions. Consequently, the adequacy of the agency’s obligation to provide explanations may be insufficient when individuals are preparing an advance directive for life-sustaining treatment (16). Knowledge about life-sustaining care was the most influential factor affecting the intention to complete ADs (17). However, other factors also play a role, including demographic and socioeconomic factors (e.g., age, religion, education, and economic level), physical health-related factors (presence of chronic or critical diseases, subjective health status), awareness of dying-well, and attitude toward Ads. These factors have all been shown to affect the ADs completion (17–19). According to a previous study, the fear that discussing AD itself might signal patients to give up hope could be a key reason healthcare providers delay important conversations (20). Contrary to concerns, empirical research has shown that completing ADs does not decrease hope in patients; notably, it has been observed to increase hope (21). While multiple factors influence ADs completion, comprehensive studies have been relatively scarce.

## 2 Objective

Therefore, this study aimed to explore the factors influencing ADs completion among older adults in Korea. This study assumed that general characteristics, physical health-related characteristics, psychological characteristics, and attitudes toward death would significantly impact the ADs completion among older adults in Korea.

## 3 Materials and methods

### 3.1 Study design

This study was a secondary analysis of the data from a previous cross-sectional study that explored the influencing factors of ADs among older adults living in the community.

### 3.2 Data collection and participants

This study analyzed the cross-sectional nationwide data obtained from the 2020 National Survey of Older Koreans, which is conducted every 3 years by the Korean Ministry of Health and Welfare. The 2020 original survey data were collected by 169 trained interviewers using the Tablet-PC Assisted Personal Interview (TAPI) method from September 14 to November 20, 2020. The interviewers selected were individuals experienced in conducting similar surveys. Furthermore, training was provided six times (22). This study was conducted via face-to-face interviews. The target population comprised older adults (65 years or above) who lived in communities in 17 cities and provinces across the country. The 2020 National Survey of Older Koreans sample was selected using a proportional two-stage stratified sampling method. First, the population was stratified by 17 metropolitan cities and provinces across Korea, and thereafter, re-stratified by neighborhood in the nine provinces (but not in the seven metropolitan cities). The Ministry of Health and Welfare research team applied various weights in the raw data to ensure the accuracy of estimations. The weight of the raw data was adjusted by considering the weights for households and individuals (22). The study population represented Korean older adults aged 65 years living in the community. Of the total 10,097 respondents from the 2020 National Survey of Older Koreans, 9,920 were selected; 167 were excluded because they comprised proxy respondents and 10 were excluded because of missing responses. The researchers received coded data, which were used for secondary data analysis.

### 3.3 Ethical considerations

The 2020 National Survey of Older Koreans was approved by Statistics Korea (Approval No. 117071). The institutional review board approval was obtained from the Korea Institute for Health and Social Affairs (IRB No. 2020–36) prior to conducting the survey. All participants provided written informed consent prior to participation and were informed that they could withdraw their consent at any time without any disadvantage. After obtaining approval for our study, we received the raw data without personal identification information. Additionally, the study was approved by the Institutional Review Board of G University (IRB No. 1044396-202202-HR-039-01)—the corresponding author is affiliated with this university. All methods were applied in accordance with the relevant guidelines and regulations.

### 3.4 Measurements

#### 3.4.1 ADs

ADs were measured using the question, “Did you complete an AD in preparation for death at the end of your life?” The response options were: 1 = yes, 2 = no.

#### 3.4.2 Sociodemographic characteristics

General characteristics (e.g., age, sex, spouse, spouse health status, religion, and education level) were self-reported.

#### 3.4.3 Physical-health-related characteristics

Physical-health-related characteristics included the presence of chronic diseases (e.g., number of diagnosed chronic diseases, medication, used medical institution, etc.), alcohol intake (frequency for 1 year), smoking (yes or no), nutritional status, body mass index (BMI), activities of daily living (ADL), instrumental activities of daily living (IADL), exercise (yes or no), muscle power, and sensory discomfort (feeling in daily life: comfortable, a little uncomfortable, and uncomfortable). Moreover, fall experience for 1 year (yes or no) was estimated.

Nutritional status was investigated using a nutritional screening initiative checklist (23). This tool comprised 10 items, and the total score was calculated out of 21 points by weighting each item. The subjects were instructed to sit on a chair or bed and then stand up five times, and the muscle power was evaluated as: performed, failed, impossible to perform, and rejected. Additionally, sensory discomforts such as hearing, vision, and chewing difficulties were investigated.

#### 3.4.4 Psychological characteristics

Psychological characteristics including depression, cognitive function, subjective satisfaction (health, economy, and overall), suicide intention, and social relationship (number of friends or neighborhood) were self-reported. Depression was estimated using the Korean version of the short form of the Geriatric Depression Scale (SGDS-K) (24), and cognitive function was estimated using the Korean version of the Mini-Mental State Examination for dementia screening (KMMSE-DS) (25).

#### 3.4.5 Attitude toward death

Attitude toward death included the attitude toward medical care for life prolongation, death preparation education (yes or no), and awareness of dying-well. The awareness of the significance of dying-well at the end of life among the older adult was estimated by adding the total of four items: preparing for dying by oneself, dying without pain, dying in the presence of family or close friends, and dying that is not a burden to family or close friends. These items were rated on a 5-point Likert scale. The lower the total score, the higher the awareness of the significance of dying-well.

### 3.5 Data analysis

The collected data were analyzed using the SPSS Win 23.0 program (SPSS, IBM Corp., Armonk, NY, United States), with the two-tailed significance level set at 0.05. The items “not applicable” and “not respond” replies were excluded from the final analysis. Differences in participants’ characteristics based on ADs completion were examined with a t-test or chi-squared test. Furthermore, the multinomial logistic regression analysis was performed to identify the predictive factors of complete ADs.

## 4 Results

### 4.1 Differences in subjects’ characteristics based on the advance directives completion

Spouse health status with a somewhat unhealthy or unhealthy state (*p* = 0.018) and religion (*p* = 0.017) were significantly prevalent in the ADs group compared to the non-ADs group. The education level was higher in the ADs group than in the non-ADs group (*p* < 0.001; Table 1). The number of chronic diseases, number of prescription medications, frequency of alcohol consumption for 1 year, nutritional status, and frequency of exercise were higher in the ADs group than in the non-ADs group (*p* < 0.001, *p* < 0.001, *p* = 0.004, *p* = 0.006, and *p* = 0.04, respectively; Table 1). Compared to the non-ADs group, the ADs group had weaker muscle power, a higher degree of depression, more disagreement with medical care for life prolongation, higher death preparation education experience, lower awareness of dying-well, and a higher level of suicide intention (*p* = 0.043, *p* = 0.004, *p* < 0.001, *p* < 0.001, *p* = 0.004, and *p* < 0.001, respectively; Table 1). Regarding the comparison of the details of dying-well awareness, the older adults in the non-ADs group considered dying without pain, dying in the presence of family or close friends, and dying that is not a burden to them as more significant than those in the ADs group (*p* = 0.032, *p* = 0.006, and *p* = 0.001, respectively; Table 1).

**Table 1:** Differences in subjects’ characteristics based on the signing of advance directives (*N* = 9,920).

Categories	Variable		ADs group (*N* = 421) Mean ± SD/N(%)	Non-ADs group (*N* = 9,499) Mean ± SD/N(%)	t/χ^2^	*p*
General characteristics	Age Sex Spouse	Men Yes	73.80 ± 6.33 207 (49.2) 262 (62.2)	73.42 ± 6.54 3,764 (39.6) 5,586 (58.8)	1.147 15.295 1.956	0.251 <0.001 0.172
	Spouse health status	Very healthy Healthy Moderate Somewhat unhealthy Unhealthy	15 (5.7) 149 (56.9) 53 (20.2) 35 (13.4) 10 (3.8)	329 (5.9) 2,960 (53.0) 1,569 (28.1) 621 (11.1) 107 (1.9)	11.936	0.018
	Religion (Yes) Education level	Yes	272 (64.6) 9.11 ± 4.10	5,578 (58.7) 8.15 ± 3.99	5.773 4.827	0.017 <0.001
Physical-health-related characteristics	No. of chronic diseases No. of prescription medications Use of medical institutions Drinking alcohol Smoking Nutritional status BMI ADL IADL Exercise	Yes Yes	2.09 ± 1.58 2.05 ± 1.63 298 (70.8) 1.42 ± 1.88 48 (11.4) 22.53 ± 1.65 23.47 ± 2.73 7.23 ± 1.20 10.69 ± 2.88 245 (58.2)	1.82 ± 1.46 1.76 ± 1.53 6,531 (68.8) 1.14 ± 1.75 1,041 (11.0) 22.76 ± 1.69 23.58 ± 2.59 7.17 ± 1.09 10.60 ± 2.58 4,942 (52.0)	3.648 3.781 0.774 2.926 0.081 −2.772 −0.816 0.958 0.647 6.148	<0.001 <0.001 0.390 0.004 0.750 0.006 0.415 0.338 0.518 0.014
	Muscle power	Performed Fail Impossible to perform Reject	310 (73.6) 77 (18.3) 16 (3.8) 18 (4.3)	6,975 (73.4) 1902 (20.0) 180 (1.9) 442 (4.7)	8.142	0.043
Psychological characteristics	Depression Cognitive function		3.84 ± 3.35 24.29 ± 6.59	3.35 ± 3.40 24.32 ± 5.25	2.888	0.004
	Subjective satisfaction (Health)	Very satisfied Satisfied Somewhat Unsatisfied Not at all	23 (5.5) 208 (49.4) 113 (26.8) 67 (15.9) 10 (2.4)	441 (4.6) 4,540 (47.8) 2,897 (30.5) 1,385 (14.6) 236 (2.5)	3.086	0.544
	Subjective satisfaction (economy)	Very satisfied Satisfied Somewhat Unsatisfied Not at all	33 (7.8) 159 (37.8) 168 (39.9) 53 (12.6) 8 (1.9)	706 (7.4) 2,994 (31.5) 3,968 (41.8) 1,611 (17.0) 220 (2.3)	10.272	0.036
	Subjective satisfaction (overall)	Very satisfied Satisfied Somewhat Unsatisfied Not at all	21 (5.0) 207 (49.2) 170 (40.4) 22 (5.2) 1 (0.2)	397 (4.2) 4,515 (47.5) 3,899 (41.0) 644 (6.8) 44 (0.5)	2.799	0.592
	Suicide intention	Yes	19 (4.5)	168 (1.8)	16.417	<0.001
	Social relationship		2.86 ± 2.41	3.05 ± 2.56	−1.512	0.131
Attitude toward death	Attitude toward medical care for life prolongation	Very agree Agree Somewhat Disagree Very disagree	0 (0.0) 0 (0.0) 44 (10.5) 201 (47.7) 176 (41.8)	9 (0.1) 221 (2.3) 1,081 (11.4) 3,703 (39.0) 4,485 (47.2)	20.873	<0.001	
Take death preparation education	Yes	63 (15.0)	206 (2.2)	250.194	<0.001		
Awareness of well-dying	Total	7.02 ± 2.19	6.70 ± 2.18	2.915	0.004		
(1) Prepare for dying by oneself	Very important Important Somewhat Not important Not at all	163 (38.7) 211 (50.1) 43 (10.2) 4 (1.0) 0 (0.0)	3,661 (38.5) 4,738 (49.9) 981 (10.3) 111 (1.2) 8 (0.1)	0.533	0.970		
(2) Dying without pain	Very important Important Somewhat Not important Not at all	215 (51.1) 148 (35.2) 52 (12.4) 6 (1.4) 0 (0.0)	5,217 (54.9) 3,399 (35.8) 795 (8.4) 80 (0.8) 8 (0.1)	10.560	0.032		
(3) Dying in the presence of family or close friends	Very important Important Somewhat Not important Not at all	133 (31.6) 216 (51.3) 63 (15.0) 7 (1.7) 2 (0.5)	3,787 (39.9) 4,425 (46.6) 1,108 (11.7) 162 (1.7) 17 (0.2)	14.478	0.006		
(4) Dying that is not a burden to family or close friends	Very important Important Somewhat Not important Not at all	153 (36.3) 214 (50.8) 54 (12.8) 0 (0.0) 0 (0.0)	4,285 (45.1) 4,310 (45.4) 854 (9.0) 46 (0.5) 4 (0.0)	18.258	0.001

ADs, advance directives; SD, standard deviation; No, number; BMI, body mass index; ADL, activity daily living; IADL, instrumental activity daily living.

### 4.2 Differences in subjects’ diagnosed chronic diseases based on the advance directives completion

The prevalence rate of diabetes mellitus (DM) was lower in the ADs group than in the non-ADs group (*p* < 0.010). The fracture experience and prevalence rate of insomnia were higher in the ADs group than in the non-ADs group (*p* = 0.024 and *p* = 0.003, respectively). The prevalence rate of cataracts and fall experience for 1 year was higher in the ADs group than in the non-ADs group (*p* = 0.003 and *p* = 0.002, respectively; Table 2).

**Table 2:** Differences in subjects’ diagnosed chronic diseases based on the signing of advance directives (*N* = 9,920).

Variable	ADs group (*N* = 421) N(%)	Non-ADs group (*N* = 9,499) N(%)	*χ^2^*	*p*
Stroke (Yes) Dyslipidemia (Yes) Angina or MI (Yes) CHF or Arrhythmia (Yes) DM (Yes) Fracture (Yes) COPD (Yes) Asthma (Yes) Depression (Yes) Parkinson (Yes) Insomnia (Yes) Cataract (Yes) Cancer (Yes) Fall experience for 1 year (Yes)	14 (3.3) 85 (20.2) 24 (5.7) 21 (5.0) 79 (18.8) 11 (2.6) 5 (1.2) 10 (5.7) 6 (1.4) 4 (1.0) 17 (4.0) 34 (8.1) 12 (2.9) 43 (10.2)	372 (3.9) 1,604 (16.9) 425 (4.5) 430 (4.5) 2,297 (24.2) 117 (1.2) 115 (1.2) 165 (1.7) 131 (1.4) 44 (0.5) 170 (1.8) 407 (4.3) 152 (1.6) 590 (6.2)	0.376 3.115 1.403 0.198 6.494 6.037 0.002 0.948 0.006 1.985 11.018 13.641 3.875 10.811	0.698 0.085 0.230 0.632 0.010 0.024 1.000 0.339 0.831 0.145 0.003 0.001 0.074 0.002

ADs, advance directives; MI, myocardial infarction; CHF, congestive heart failure; DM, diabetes mellitus; COPD, chronic obstructive pulmonary disease.

### 4.3 Differences in sensory discomfort based on the advance directives completion

The rate of feeling a little uncomfortable in terms of hearing, vision, or chewing was higher in the ADs group than in the non-ADs group (*p* = 0.010, *p* = 0.001, and *p* = 0.036, respectively; Table 3).

**Table 3:** Differences in sensory discomfort based on the signing of advance directives (*N* = 9,920).

Variable		ADs group (*N* = 421) N(%)	Non-ADs group (*N* = 9,499) N(%)	*χ^2^*	*p*
Hearing	Comfortable A little uncomfortable Uncomfortable	313 (74.3) 106 (25.2) 2 (0.5)	7,310 (77.0) 1986 (20.9) 203 (2.1)	9.232	0.010
Vision	Comfortable A little uncomfortable Uncomfortable	248 (58.9) 164 (39.0) 9 (2.1)	6,386 (67.2) 2,911 (30.6) 202 (2.1)	13.154	0.001
Chewing	Comfortable A little uncomfortable Uncomfortable	254 (60.3) 158 (37.5) 9 (2.1)	5,915 (62.3) 3,178 (33.5) 406 (4.3)	6.631	0.036

ADs, advance directives.

### 4.4 Associated factors of the advance directives completion

A multiple logistic regression analysis was conducted to examine the factors affecting the advance directives completion. The analysis included variables with significant differences in the advance directives completion by univariate analysis (sex, religion, education level, number of chronic diseases, alcohol consumption frequency for 1 year, nutritional status, depression, subjective satisfaction with the economy, attitude toward medical care for life prolongation, awareness about dying-well, DM, fracture, insomnia, fall experience over 1 year, death preparation education, suicide intention, vision discomfort, hearing discomfort, and chewing discomfort). Before the analysis, the Hosmer and Lemeshow test was conducted to identify the goodness of fit. Our model could be considered reasonably good because *p* = 0.455. The associated factors of the advance directives completion included men, higher education level, exercise, death preparation education, lower awareness of dying-well, and experience of fracture (*p* = 0.003, *p* = 0.009, *p* = 0.036, *p* < 0.001, *p* = 0.001, *p* = 0.004, respectively; Table 4). Moreover, the odds ratio of completion of advance directives was significantly lower among participants with DM or moderate health spouses compared to those with very unhealthy spouses (*p* = 0.001, and *p* = 0.015, respectively; Table 4).

**Table 4:** Associated factors of the signing of advance directives (*N* = 9,920).

Independent variable		*B*	*SE*	*p*	Adjusted OR	95% CI	
						Lower	Upper
Sex (men)		0.446	0.152	0.003	1.563	1.160	2.104
Spouse health status	Very healthy Healthy Moderate Somewhat unhealthy	−0.563 −0.552 −0.931 −0.576	0.465 0.371 0.382 0.395	0.226 0.137 0.015 0.146	0.570 0.576 0.394 0.562	0.229 0.278 0.187 0.259	1.416 1.191 0.832 1.221
Religion (No)		−0.112	0.138	0.419	0.894	0.682	1.172
Education level		0.052	0.020	0.009	1.053	1.013	1.096
No. of chronic diseases		0.078	0.078	0.318	1.081	0.928	1.259
No. of prescribed medications		0.067	0.058	0.249	1.070	0.954	1.199
Alcohol consumption fx for 1 year		−0.015	0.038	0.705	0.986	0.914	1.063
Nutritional status		−0.052	0.050	0.299	0.949	0.860	1.048
Exercise (Yes)		0.295	0.140	0.036	1.343	1.020	1.768
Depression		0.034	0.024	0.169	1.034	0.986	1.085
Subjective satisfaction (economy)	Very satisfied Satisfied Somewhat Unsatisfied	−0.229 −0.456 −0.266 −0.684	602 0.563 0.555 0.575	0.703 0.418 0.632 0.234	0.795 0.634 0.766 0.504	0.245 0.210 0.258 0.164	2.586 1.912 2.276 1.556
Attitude toward medical care for life prolongation	Very agree Agree Somewhat Disagree	−19.272 −18.849 −0.470 −0.090	15896.291 3410.416 0.245 0.146	0.999 0.996 0.055 0.539	0.000 0.000 0.625 0.914	0.000 0.000 0.387 0.687	. . 1.010 1.217
Death preparation education (yes)		2.123	0.212	0.000	8.359	5.517	12.663
Awareness of well-dying		0.109	0.032	0.001	1.115	1.046	1.188
Suicide intention (yes)		0.676	0.400	0.091	1.965	0.897	4.306
DM (yes)		−0.660	0.193	0.001	0.517	0.354	0.755
Fracture (yes)		1.235	0.433	0.004	3.438	1.472	8.027
Insomnia (yes)		0.622	0.411	0.130	1.862	0.832	4.169
Cataract (Yes)		0.484	0.265	0.068	1.623	0.965	2.730
Fall experience over 1 year (yes)		0.006	0.296	0.983	1.006	0.563	1.799
Hearing discomfort	Comfortable A little uncomfortable	17.711 17.756	4004.037 4004.037	0.996 0.996	49177412.172 51448667.699	0.000 0.000	
Vision discomfort	Comfortable A little uncomfortable	0.399 0.653	0.787 0.782	0.612 0.404	1.490 1.921	0.319 0.415	6.963 8.898
Chewing discomfort	Comfortable A little uncomfortable	2.073 1.865	1.036 1.031	0.045 0.070	7.948 6.456	1.044 0.856	60.532 48.687
	Nagelkerke R^2^ = 0.128						

SE, standard error; OR, odds ration; CI, confidence interval; No, number; fx, frequency; DM, diabetes mellitus.

## 5 Discussion

In Korea, the proportion of older adult is rapidly increasing because of the low birth rate, the aging population, and improvement of medical technology (26). Although the average lifespan has increased, new challenges are emerging—for example, burden on individuals and society because of life-sustaining treatment which only prolongs the process of dying for the older adults with no possibility of recovery (27). With increasing societal interest in patient autonomy and a dignified and comfortable end of life, ADs for life-sustaining treatment were introduced in February 2018 to allow patients to end their lives with dignity (28).

From 2018 to 2021, a total of 194,181 cases were actually utilized of life-sustaining treatment plans for the entire population in Korea; however, only 7.5% of them were written by the patients themselves (29). The proportion of ADs signed by patients has been increasing every year since 2018. This study attempted to identify the ADs documentation rate in older people in Korea and investigate its influencing factors by using the data from the 2020 National Survey of Older Koreans (22).

The current ADs completion rate remains low at 4.24% among people aged 65 years and above. In some eastern countries, such as Japan, China and Hong Kong, advance directives are still very slowly being promoted, but they are not legally enforceable because people generally avoid talking about death because it is considered disrespectful (30, 31). The ADs documentation system is known to be effective in patient care (32), decreased healthcare cost and utilization (33), and decreased futile life-sustaining treatment (34). Nevertheless, even in the United States, where relevant legislation was enacted even earlier, the rate of completed ADs was reported to be 26% in 2009 and 2010 (35). Whites are more likely to complete AD than Blacks or Hispanics (36). Previous studies have shown that this is because racial/ethnic differences in complete AD are multifaceted, strongly influenced by a variety of cognitive and psychological, sociocultural, and sociodemographic factors (37). Taken together, race/ethnicity can be seen as a proxy for personal, cultural, and social contexts, so that an individual’s values, beliefs, and personal circumstances are necessary for the completion of ADs.

This study investigated the factors affecting ADs documentation among older adults, including general characteristics, physical-health-related characteristics, psychological characteristics, and attitudes toward death. The significant factors that increase the ADs group’s odds ratio compared to the non-ADs group included sex, education level, spousal health status, exercise, death preparation education, and fracture experience. The lowering factors included the absence of difficulty in chewing, presence of DM, and awareness of dying-well.

The ADs completion rates were 5.2% for men and 3.6% for women. The odds ratio for men to belong to the ADs group was higher than for women. This result contradicted the previous report that women had a higher ADs completion rate than men in all age groups in Korea (29). This discrepancy may be because the participants of this study included only the community-dwelling older adults with a high proportion of men and excluded the older adults in medical facilities (22). In a previous study among older Korean-American adults, sex was not a significant factor in ADs completion, even though ADs awareness was higher in women than men (38). Future research should examine the differences this study found by attempting to determine whether there are gender differences in ADs documentation, including among older adults admitted to facilities and hospitals.

The result that death preparation education is an influencing factor of ADs completion is consistent with the results of previous studies that reported that direct patient-healthcare professional interactions may be more effective than passive patient education materials (39). However, lower awareness of dying-well an influencing factor of ADs completion was an unexpected result. According to a previous study, people tend to make decisions based on the values and possibilities inherent in a specific situation (40). Another study found that awareness of ADs and higher education level were significant factor in ADs completion (37, 41). Although the number of registered agencies for advance directives are increasing (28), it is difficult to find an agency that helps older adult prepare for death. Therefore, efforts to increase the awareness of dying-well and the significance of end-of-life planning are required.

The number of chronic diseases, number of prescribed medications, depression, insomnia, and suicide intention were higher in ADs group compared to the non-ADs group. These results are consistent with the results of a previous study that participants with poorer health status are more willing to complete ADs (19). The finding that older adults with higher levels of depression, insomnia, and suicide intention are more likely to sign ADs indicates the need to focus on the mental health of older adults. Self-determining ADs should facilitate a dignified dying; therefore, it should be done in a healthy mental and cognitive state that could fully contemplate one’s dying with dignity.

Moreover, the spouse’s and the participant’s health status were significant influencing factors. In this study, the OR of ADs completion was lower in the normal group compared to the group in which the spouses’ health status was reported as poor. In a previous study, severe spousal illness and illness severity were strongly correlated with ADs completion (42). Other chronic diseases did not significantly impact the signing of ADs; DM was a lowering factor, and fracture experience was a significant factor in signing ADs. These results may be because older adults have more opportunities to consult about ADs in a healthcare facility while hospitalized for fractures (43). Furthermore, fractures affect hundreds of thousands of older adults yearly (44). Severe complications may occur after surgeries, a hospitalization period is long, and a substantial proportion of patients will die within months of the injury (40, 41). Therefore, it is vital for patients older than 65 years undergoing these fractures to have a system that facilitates ADs completion (45).

### 5.1 Limitations

This study has several limitations. First, the secondary data analysis used only the variables included in the raw data. Although efforts were made to approach them as comprehensively as possible, it was challenging to identify factors that were not included in the raw data. Therefore, there are potential variables in the experimental research, including the study, that should be conducted in the future. In particular, discussions on good death, end of life, autonomy, and AD writing should be based on a variety of social and cultural backgrounds, but this paper did not explore these cultural backgrounds in depth. Therefore, we recommend future research to explore the cultural characteristics of AD writing among Koreans. Second, this study included only those community-dwelling older adults who could self-report. Thus, study findings are not generalized to older adults who are institutionalized or admitted. Future studies should include older adults admitted to a facility. Third, owing to cross-sectional secondary data, causality could not be explored; this should be clarified in a future longitudinal study.

## 6 Conclusion

The ADs documentation rate remains low among those aged 65 years and above. The comparison of an ADs group with a non-ADs groups in this study revealed that various general, physical, and psychological characteristics influenced the advance directives completion. Although ADs aim to reduce unnecessary medical treatment and facilitate a dignified death, the rate of ADs completion remains rather low among the older adults with high awareness of dying-well. Therefore, information dissemination regarding ADs should be promoted and relevant authorities should consider multiple options to improve the physical and psychological health of older adults, as well as their attitude toward death to increase the ADs completion rate. Furthermore, it is essential to devise creative and cost-effective person-to-person intervention strategies considering the importance of participants’ ability to ask questions and receive assistance with completing ADs.

## Data availability statement

The raw data supporting the conclusions of this article will be made available by the authors, without undue reservation.

## Ethics statement

The studies involving humans were approved by Institutional Review Board of Gachon University. The studies were conducted in accordance with the local legislation and institutional requirements. The participants provided their written informed consent to participate in this study.

## Author contributions

SC: Conceptualization, Methodology, Software, Supervision, Validation, Writing – original draft, Writing – review & editing. HK: Conceptualization, Methodology, Validation, Visualization, Writing – original draft, Writing – review & editing.
